# Lived experiences of stressors and problems of higher education students on teacher education course in the Eastern Highlands of Zimbabwe, 2019

**DOI:** 10.11604/pamj.2020.36.289.21481

**Published:** 2020-08-17

**Authors:** Mathew Nyashanu, Rebecca Nuwematsiko, Wendy Nyashanu, Dung Ezekiel Jidong

**Affiliations:** 1 Nottingham Trent University, 50 Shakespeare St, Nottingham NG1 4FQ, United Kingdom,; 2 Makerere University School of Public Health, College of Health Sciences, Kampala, Uganda,; 3 Birmingham and Solihull Mental Health Foundation Trust NHS, 1, B1, 50 Summer Hill Rd, Birmingham B1 3RB, United Kingdom

**Keywords:** Lived experiences, stressors, students, higher education, Zimbabwe

## Abstract

**Introduction:**

there is increasing levels of stressors and hardship among higher education students especially in low and middle income countries. Higher education institutions have an important role to play in the provision of robust and comprehensive support for students who experience stressors and hardship. Research and action in this area has however not been prioritized by the institutions in Zimbabwe. This study examined students´ expression of their experience with stressors and problems of studying in higher education in the Eastern Highlands of Zimbabwe.

**Methods:**

the study employed a qualitative approach using the phenomenology approach. Three institutions of higher education in the eastern border highlands of Zimbabwe were considered. Four focus group discussions were conducted with eight participants in each group. A one-to-one semi-structured interview with eight individual participants was also conducted to further examine the issues raised in the focus groups. Data were analyzed thematically using the Silences Framework theoretical model.

**Results:**

five overarching themes emerged from the analysis: (i) the stress of completing assessments without adequate learning materials. (ii) Unfair placement workload results into poor assessment outcomes. (iii) College-life is more difficult due to financial constraints. (iv) Marital problems interfering with college work: there is no mental health service available. (v) Enduring pains of bereavement with no emotional support or helpline.

**Conclusion:**

the study recommends the need to develop an inter-ministerial mental health strategy for institutions of higher learning with the view of implementing policies that address students suffering in Zimbabwean HE institutions.

## Introduction

Higher education (HE) institutions have an important role to play in the provision of robust and comprehensive support for students who experience stressors and hardship [1, 2]. Stressors associated with HE may partly explain current increase in the number of students declaring mental health conditions such as suicidal ideations [3-5]. Suicidal ideations might be developed as a result of depression and anxiety which are more prevalent among students in HE as compared with the general population [6, 7]. In the context of this study, the impact of lived stressors and mental health will be used interchangeably. Since the publication of the document Learning Together to Work Together by the World Health Organization [8], national and international policy and research institutions have called for greater inter-professional cooperation and collaboration between staff working in health and social care services including higher education to provide a wholesome approach to the prevention of increasing stressors and hardship among higher education students [9-14]. Inter-professional collaboration has been advocated as a means of enhancing the quality of life and positive creation of choice for support to the alleviation of in the higher education.

There are many problems and stressors that higher education students come with from their diverse backgrounds, which are often underestimated [15, 16]. These problems are rarely given priority, especially if they are not related to the outcomes of the course the students are taking. Many higher learning institutions are focused on achieving the key aims and objectives laid down by the governing bodies and the Ministry of Higher Education without paying due attention to the needs of the students. Furthermore, higher education institutions are now realising the need to establish mental health and counselling services within their institutions in order to provide substantial help and support for their students [17]. Higher education institutions in Low and Middle Income Countries (LMICs) are faced with a myriad of problems, ranging from lack of skilled staff in various fields to financing of workers [15]. This has affected the availability of some services such as mental health support with live stressors which are desperately needed but are highly stigmatised by the communities [18]. The stigmatisation of mental health has also silenced many scholars and government departments from taking a proactive stance in enhancing the welfare of affected individuals. The socially constructed stance by communities that viewed mental health as the fault of the affected individuals has also prevented some individuals from communicating their problems [19].

Many LMICs lack robust strategies that cut across different institutions to reach out for all individual students, including those who cannot present themselves at a conventional support system or mental health treatment centre [20]. Lack of financial support to students compounded with issues such as unresolved marital problems may have a substantive impact on mature students achievements [21]. More so, there is very limited inter-ministerial work to combat sources of stress or mental health problems in many developing countries [22]. Most of this work has been left to the third sector and religious organisations who have tried to fix the problem with a ‘one size fits all’ approach. Such an approach has also been marred by unorthodox interventions that have left communities at crossroads with accusations and counter-accusations of witchcraft [23]. Zimbabwe's education system has been hit the hardest following many years of economic and political turmoil. Parents have struggled to send their children to university and most of the time they just manage to fund fees and accommodation and then students have to find a way to obtain the basic needs. This struggle to attain education and basic needs while at school has contributed to the stressors among Zimbabwe students consequently affecting their mental health. A typical university or higher education college in Zimbabwe will have, at most, four trained student counsellors dealing with at least 15,000 students; equivalent to one counsellor per 3,750 students. Despite availability of the counselling services, literature shows that most students don´t approach counselling departments to get help when they experience anxiety, stress or depression, or when they feel suicidal [24]. It is against this background that the present study examined lived experiences of stressors and problems of higher education students in the Eastern Highlands of Zimbabwe.

## Methods

The study was conducted in higher education institutions in the Eastern Highlands of Zimbabwe between January and February 2019 employing a qualitative approach using the descriptive phenomenology methodology. Qualitative research is a paradigmatic with its origins in socio-psychology which gives a holistic description of a social context through the worldview of the participants [25-27]. According to this definition, the emphasis is placed on the meaning of social phenomena as viewed by those involved in particular contexts (Lived experiences) [28]. The method gives scope for describing events from a lived experience perspective. In this study stressors are viewed as any events, experiences, or environmental stimuli that cause stress among students. These events or experiences are perceived as threats or challenges to the students in higher education and can be either physical or psychological. It was imperative that this social group was studied owing to lack of attention to mental health and well-being of students in higher education across the world and specifically in Zimbabwe.

Four focus group discussions (FGDs) were conducted among students undertaking teacher education from three institutions of higher education in the Eastern border highlands of Zimbabwe to understand the student´s lived experiences of stressors and problems while in higher education. Each FGD comprised of eight participants with equal gender balance and lasted approximately 60 minutes. In order to triangulate the data, eight one-to-one follow-up semi-structured interviews which lasted approximately 30 minutes were also conducted among the students to further examine the issues raised in the focus groups. In order to ensure rigor, the procedure consisted of two follow-up interviews after each FGD. A purposive sampling technique was used to select participants for this study. All participants were identified through the Fellowship of College Students (FOCUS) and sign language clubs in the Eastern Highlands of Zimbabwe. Participation was on a voluntary basis, with participants responding to issues concerning their mental well-being at different institutions. More so, all participants completed a consent form after reading an information sheet. The participants were also aware that they could withdraw from the study at any time without giving a reason. Ethical approval to conduct the study was granted by the research and development unit.

**Figure 1:**
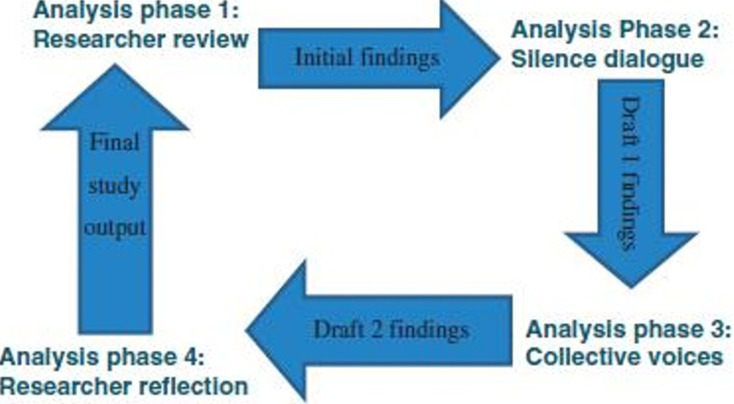
representation of the phases of data analysis: findings from a study on lived experiences of stressors and problems of Higher Education students in the Eastern Highlands of Zimbabwe, 2019

All interviews and focus group discussions were audio recorded and later transcribed verbatim. The interview transcripts were analysed thematically using the Silences Framework model (Figure 1) [29]. Excerpts and direct quotes were used to present evidence for the themes emerging from the analysis. The Silences Framework model was adopted because it is suitable for researching sources of stressors in HE that are little-researched. It projects both the dominant and marginalized views of communities and exposes the multiple realities that exist within a group or society [30]. The Silences Framework asserts that reality is not objective or fixed, but rather human beings are the script authors of the social world in any society at a particular time [29]. The Framework puts more emphasis on the individual or group interpretation of events and human experiences, which can be viewed as their truth.

### The phases of data analysis

As shown in Figure 1, the data were subjected to the four phases of analysis in line with the theoretical framework guiding this study. This enabled data triangulation and enhanced the quality of the results obtained as detailed below. In phase 1, data collected was first analysed by the researchers with reference to the research questions including the acknowledged limitations. The research participants in phase 2 generated the findings from this analysis for further scrutiny and verification. Phase 2 entailed an advance review of preliminary analysis of findings from phase 1 to produce a silence dialogue across the datasets. This was used as a benchmark to interrogate, refute or ratify the findings from the study at this stage. This was iterative and took the form of feedback and confirmation to enhance further deliberations on the findings. The researchers then revisited the initial findings in phase 1 to incorporate detailed second level analysis in phase 2. The phase 3 stage emerged after the production of draft 1 findings in the above two phases, further analysis was expanded to include social networks of research participants and others who impacted on the study but did not take part. These significant individuals were group of students who mirrored the diverse social network of the participants. This rigor is essential for the study´s applicability and transferability of findings and recommendations to similar population. Finally, in phase 4, the researchers reflected on the findings of phase 3 by revisiting, reviewing and developing emerging themes, which were taken as the final output of the research study as discussed in the next section.

## Results

**The stress of completing assessments without adequate learning materials:** the students indicated that the lecturers who seemed more concerned with the students meeting the deadlines for the assignments were subjecting them to pressure of work. They felt that the psychosocial side of their life was not being addressed. They reported feeling frustrated and unsupported with their concerns, which they could not communicate to anyone due to the stigma associated with anything to do with mental health problems due to hardship. One interviewee said: *“All lecturers expect you to hand in assignments on time, which put us under a lot of pressure to beat deadlines. The authorities seem to be more concerned with us fulfilling the college outcomes. I really feel unsupported and frustrated when there is no one to tell your concerns. More so, it is difficult to share anything to do with mental health because of the stigma associated with it” (accounts student)*. Furthermore, the students blamed the scarcity of learning resources such as books and laptops, which made it more difficult to complete assessment on time. They found it difficult to work away from the library and bemoaned the shortage of up-to-date resource materials, leading to a scramble for books to meet the deadline for their assignments.

**Unfair placement workload results into poor assessment outcomes:** many students felt that while they were on placement, their mentors were not serving them well enough as they piled a lot of work on the students to leave themselves with time to do other things not related to their teaching roles. Those on teaching placement felt that they were being subjected to stress when the mentors were demanding high standards from student teachers working with meager resources. Some students felt that it was unfair for their mentors to use pass rates as the standard for measuring their ability despite the fact that there were many other factors that contributed to the failure rates of pupils. One of the interviewee said: *Some of these mentors offload all their work to the student teachers, which becomes a burden because as a student teacher you are expected to be up to date with your work. They would leave you in charge of the class while they do other things, which have nothing to do with teaching. The other depressing thing is that some mentors use the pass rates of the class to judge your competence yet there are so many factors, which contribute to class perform (engineering student)*. The students felt that they were being trapped from both sides as the mentor had more influence on what the lecturers would write about them in their final assessment. Some students reported a culture of bullying from mentors as they purported to be holding a lot of power in deciding the final destiny of the students with regard to their final assessment while on placement. Some students confirmed that they broke down mentally but continued to work without seeking help.

**College-life is more difficult due to financial constraints:** most of the students reported that they were stressed due to a shortage of money to pay tuition fees following a protracted struggle to secure a place at the college. Some of the students reported going for days without a proper meal in order to save money for the fees. They reported that they felt weak and unmotivated due to the quality of life they were experiencing at the college. For example, a student-teacher said: *I really feel stressed that you have to struggle each term to secure fees to pay, while on the other side you really struggled to secure the place at the college. I sometimes go for days without having a proper meal because I want to save money to pay fees. The quality of life at college is really unbearable” (student teacher)*. Furthermore the students reported that there were no other sources of money especially when they had to be in class every day, and that the economic outlook made it very difficult for them to secure temporary jobs to supplement their finances. The problem was compounded with incidences of sexual harassment experienced by female students from older males who took advantage of their plight.

**Marital problems interfering with college work: there is no mental health service available:** some students reported that they were experiencing marital problems, which interfered with their college work leading to stress and possible lapses for some, who had experienced mental ill health before. The students felt that there was no scope to help anyone experiencing marital problems within their institution, as it was perceived as a secondary matter. Both male and female students described how marriage breakdown in some cases was a result of unresolved college problems taking over the students´ marital time. *It is difficult to manage a family while you are a student. Some problems that come with completion of college work can prevent you from seeing your family for weeks, ending up rocking your marriage. It is taxing when you sit down and comprehend that I lost my family because I needed a profession for life, which came with unbearable costs (business studies student)*. Most of the students felt that marriage and divorce were sensitive matters close to the heart and could not be shared with anyone. Most of the students acknowledged that they suffered in silence, as there was nowhere to seek help.

**Enduring pains of bereavement with no emotional support or helpline:** bereavement was another issue cited by students as a central problem pushing a lot of them into mental health problems. They reported that sometimes they had to deal with a situation where a close relative was being buried while they were taking a critical assessment on their course, leaving them seized by mental health problems. They bemoaned the non-availability of an empathetic service that could save them from all this turmoil. *I had to let my father go to be buried while I was not there. I could not afford to lose my degree due to non-attendance of examinations. It only takes someone to be brave enough to do so but it is a question of time because eventually you will succumb to mental health problems. It is unfortunate that this beautiful institution lacks supporting systems to help people losing their beloved ones” (computer-studies student)*. Some of the students advocated the establishment of a mental health service within the institution to provide this critical service especially to the vulnerable students. Some expressed the need for a joint ministerial intervention to come up with a mental health strategy for students in institutions of higher learning.

## Discussion

This discussion is focused on evidence that contributes to the development of existing programmes and actions within the institutions of higher education. An ample of evidence in previous studies have supported the findings of the present study which suggests that about one-third of students are likely to suffer from depressive emotional disorders and most of which goes unnoticed in many higher institutions of learning [3, 5-7, 30-33]. This state of affairs should not be ignored, as it is apparent that this emotional distress may be increasing and devastating, thereby endangering the future of college students. Most specifically, the main findings of this study revealed that exerted pressure on students affect their mental health, well-being and academic performance. The problems that were identified as stressors are wide and varied depending on the social group of students under consideration. There were emergence of Silences shared among the students which suggests that lecturers and the authorities in higher institutions of learning were either not aware or not taken into consideration. Thus, students expressed concerns about the pressure being exerted on them by the lecturers, placement mentors and the institutions´ administrators in addition to other social issues. This kind of pressure is not peculiar to students in Zimbabwe alone but has been confirmed in other diverse populations of higher education students across the world [34].

It appeared in the present study that there is a gap in the conversations between lecturers, placement mentors and students concerning other problems that are not directly related to their college and placement work. However, while the students felt that there was a need for lecturers and placement mentors to do more in helping them to deal with the pressure created by college work, they were also mindful of the need to recognise the stigma associated with seeking help for anything connected to mental health [35]. This is a deep-rooted issue entrenched in the social construction of mental health stigma within communities not only in Zimbabwe but in other LIMCs. Furthermore, the other two relevant stressors raised by the students included financial constraints and deficit in basic survival resources while in college which has resulted into some mature students failing to accomplish their professional goals. Similar findings have been recorded by Pedersen [21]. There was a strong feeling among the students that the socio-economic system had abandoned them following the world economic recession and slump in the Zimbabwean economy at the start of the new millennium, which has still not picked up [22]. Therefore, there is a need to recognise the dual responsibility placed upon students of satisfying college work requirements and looking after their welfares and families. Many scholars have acknowledged the power and effectiveness of therapy and counselling in influencing positive mental health well-being, and acclimatizing to complicated financial and marital problems [36, 37]. As such, it is imperative for colleges and work placements to provide helpful services to curb the ever-growing impact of financial and marital problems among the student population.

Our results suggest that students acknowledged some help being available in institutions of higher education but were reluctant to take them up due to stigma and gossip backlash. Confidentiality is a key factor in the successful operation of stigmatised services [38]. Mentors and lecturers need to recognise the impact that stigma has on bringing up sensitive intervention such as counseling services for mental health and marital problems in the presence of peers and friends. Confidentiality will act as a safety net for those students who are like to suffer from stigma. There is also a growing body of knowledge confirming that some students prefer to share their confidential issues with their friends rather than the conventional services available within the institutions. For example, Hawton *et al*. [39] found similar outcome which suggest that in times of stress students may confide in their fellow mates as opposed to mental healthcare service providers.

Finally, another overarching theme was bereavement among the student population. It is important to recognise that individuals are unique and have different preferences [40]. It is therefore important for higher education institutions to provide a range of optional services for students to choose, in line with the recognition of diversity and choice within the student population [41]. One of the strengths to our study is that this is the first time the sources of stressors in HE are prioritized for research in Zimbabwe. Exploring lived experiences of the higher education students is also a strength to our study since it reveals narrative experiences of the stressors and problems faced by students of higher education undertaking teaching education course. However, in order to exhaustively get a deeper understanding of the lived experiences of stressors and problems of higher education students, we included a small sample size from the three institutions; this limits the generalizability of our findings to other settings. Nonetheless, we believe findings from this study are still relevant to inform decisions and policies in similar settings in Zimbabwe given the similarity of contexts in the higher education institutions.

## Conclusion

The study reveals a need to establish a robust onsite student support system to mitigate stressors affecting students in higher education. It is also important that mental health awareness is rolled out to students including signposting them to the available facilities. When mental health of students is not attended to, it negatively affects academic and social performance and at worst it progresses to suicide. More importantly there is a need to improve the confidentiality aspects of mental health services within higher education institutions to mitigate mental health stigma and increase the uptake of mental health services among higher education students on teacher education course. Lack of confidentiality and stigma further increase the effects and challenges of mental health. In light of the issues discussed in this study, the following recommendations are made: first, an effective mental health promotion which not only attends to the needs of those affected by daily academic difficulties but also includes promoting the general well-being of all staff and students is paramount. This will bring significant benefits to the HE institutions and improve the well-being of all involved. It is imperative that the Ministry of Health in conjunction with the Ministry of Higher Education formulates a national mental health strategy to support staff and students in higher education. The policy should then be cascaded down to all institutions of higher learning, which should then make bespoke policies to suit the situations at their various institutions. Secondly, the Ministry of Higher Education needs to carry out more research and implement policy for a curriculum development that is realistically practicable for all students. Finally, there is a need to consider the creation of a service that employs qualified mental health nurses and psychologists at each institution of HE in order to cater for the students´ problems and suffering before they escalate. The established services should be mindful of the stigma that may exist among the social divide and endeavor to eradicate it through systematic and robust people-oriented mental health promotion.

### What is known about this topic

- There is an increase in the number of students declaring mental health conditions such as suicidal ideations;
- Students who receive social-emotional and mental health support achieve better academically;
- Mental health services are limited in higher education institutions in Low and Middle Income Countries which further aggravates the problem.

### What this study adds

- The study reveals a need to establish a robust onsite student support system to mitigate financial, psychological, emotional and social stressors affecting students in higher education institutions; beyond academics, students exist in a real world with socio-economic challenges which if not addressed affect their mental health and performance;
- Stressors arising from meeting strict deadlines and taking on extra assignments meant for the teaching staff have also been revealed in this study which calls for awareness and sensitisation on mental health among the teaching staff and students and capacity building for self-efficiency in tackling mental health;
- The study also emphasizes the need for mental health services in higher education institutions and improved confidentiality to mitigate mental health stigma and increase the uptake of mental health services among students.
